# ACUTE APENDICITIS IN LIVER TRANSPLANT RECIPIENTS

**DOI:** 10.1590/0102-6720201600010008

**Published:** 2016

**Authors:** Olival Cirilo Lucena da FONSECA-NETO, Heloise Caroline de Souza LIMA, Paulo Sérgio Vieira de MELO, Roberto LEMOS, Laércio LEITÃO, Américo Gusmão AMORIM, Cláudio Moura LACERDA

**Affiliations:** University Hospital Oswaldo Cruz, Faculty of Medical Sciences of Pernambuco, University of Pernambuco, Recife, PE, Brazil

**Keywords:** Appendicitis, Liver transplant, Complications

## Abstract

***Background*:**

Appendicitis is a common cause of emergency surgery that in the population undergoing organ transplantation presents a rare incidence due to late diagnosis and treatment.

***Aim*:**

To report the occurrence of acute appendicitis in a cohort of liver transplant recipients.

***Methods*:**

Retrospective analysis in a period of 12 years among 925 liver transplants, in witch five cases of acute appendicitis were encountered.

***Results*:**

Appendicitis occurred between three and 46 months after liver transplantation. The age ranged between 15 and 58 years. There were three men and two women. The clinical presentations varied, but not discordant from those found in non-transplanted patients. Pain was a symptom found in all patients, in two cases well located in the right iliac fossa (40%). Two patients had symptoms characteristic of peritoneal irritation (40%) and one patient had abdominal distention (20%). All patients were submitted to laparotomies. In 20% there were no complications. In 80% was performed appendectomy complicated by suppuration (40%) or perforation (40%). Superficial infection of the surgical site occurred in two patients, requiring clinical management. The hospital stay ranged from 48 h to 45 days.

***Conclusion*:**

Acute appendicitis after liver transplantation is a rare event being associated with a high rate of drilling, due to delays in diagnosis and therapy, and an increase in hospital stay.

## Abstract (pt)

***Racional*:**

Apendicite é causa comum de emergência cirúrgica, que na população de indivíduos submetidos ao transplante de órgãos possui incidência rara e atrasos no diagnóstico são frequentes.

***Objetivo*:**

Relatar a ocorrência de apendicite aguda em uma coorte de pacientes receptores de transplante hepático.

***Método*:**

Foram analisados retrospectivamente, no período de 12 anos casuística de 925 transplantes de fígado, onde cinco casos de apendicite aguda foram encontrados.

***Resultados*:**

O aparecimento da apendicite ocorreu entre 3 e 46 meses após o transplante, a idade variou entre 15 e 58 anos; três eram homens (60%) e duas mulheres (40%). As apresentações clínicas foram variadas, mas não discordantes daquelas encontradas em pacientes não transplantados. Dor foi achado presente em todos os pacientes, sendo em dois bem localizada em fossa ilíaca direita (40%). Dois deles apresentaram sintomatologia característica de irritação peritoneal (40%) e um distensão abdominal (20%). Todos foram abordados por laparotomia. Em 20% não houve complicações e em 80% foram realizadas apendicectomias complicadas por supuração (40%) ou perfuração (40%). Infecção do sítio cirúrgico superficial ocorreu em dois pacientes tratados clinicamente. O tempo de alta hospitalar variou de 48 h a 45 dias.

***Conclusão*:**

A apendicite aguda após transplante hepático é evento raro. Associa-se com alta taxa de perfuração decorrente aos atrasos no diagnóstico e tratamento. Cursa com mais longo internamento hospitalar.

## INTRODUCTION

Appendicitis is a common cause of surgical emergency, and in the population of patients undergoing organ transplantation it is rare^3^^,^ 5 ^,^ 6 ^,^ 7 ^,^ 9 ^,^ 11. In these cases it has often clinical presentation similar to non-transplant patients. Complications from diagnostic delays are frequent, as well as the difficulty of making the differential diagnosis with other causes of acute abdomen. Only a few case reports and studies are present in literature^1^^,^ 2 ^,^ 3 ^,^ 4 ^,^ 7 ^,^ 8 ^,^ 10 ^,^ 11.

The aim of this study was to report the occurrence of acute appendicitis in a cohort of patients receiving liver transplantation.

## METHOD

Retrospectively review of 12-year period (January 2002 to December 2014) where 925 liver transplants were done with five cases of appendicitis. They were treated by the staff of the University Hospital Oswaldo Cruz Liver Transplant Unit, Recife, PB, Brazil. The selected variables were gender, age, transplant indication, post-transplant time, symptoms, surgical access, intraoperative findings and hospitalization period.

## RESULTS

The patients suffering from acute appendicitis had liver transplant indication for: Budd-Chiari syndrome; secondary biliary cirrhosis and cirrhosis due to hepatitis C; primary biliary cirrhosis and cirrhosis due to hepatitis C; and cirrhosis in alcoholic disease and hepatitis C. The MELD (Model for End-stage Liver Disease) ranged from 19 to 25. Two were transplanted in the pre-MELD era (before June 2006).

The emergence of acute appendicitis occurred from three to 46 months after liver transplantation in a population ranged from 15 to 58; were three men (60%) and two women (40%). The clinical presentations were varied, but not discordant from those found in non-transplanted patients. Pain was present in all patients, in two well located in the right iliac fossa (40%). Two patients had characteristic symptoms of peritoneal irritation (40%) and distension (20%).

All were approached by laparotomy. In 20% there were no complications and 80% had complications due to suppurative appendectomies (40%) or perforation (40%). The procedure was performed in all was appendectomy locking appendiceal stump with double suture with nonabsorbable thread, washing the cavity with saline solution 0.9%. The antimicrobials used were metronidazole associated with ceftriaxone in all, varying administration from 24 h to 60 h.

superficial surgical site infection occurred in two patients requiring clinical management. The hospital stay ranged from 48 h to 45 days (Table 1)

**TABLE 1:** - Characteristics of patients and treatments

**Caso**	**Sexo**	**Idade**	**Tempo pós-transplante**	**Sintomas**	**Via de acesso**	**Achado intra-operatório**	**Alta Hospitalar**
1	Masculino	15 anos	2 anos	Distensão abdominal	Laparotomia mediana xifo púbica	apêndice perfurado e peritonite	Alta após 45 dias
2	Feminino	49 anos	3 anos e 10 meses	Anorexia e dor em FID	Incisão transversa em FID	apendicite supurativa	Alta após 48 horas
3	Feminino	38 anos	1 ano e 7 meses	Dor em FID	Incisão transversa em FID	apendicite supurativa	Alta após 72 horas
4	Masculino	41 anos	6 meses	Peritonismo	Laparotomia infraumbilical	apendicite supurativa e peritonite	Alta após 7 dias
5	Masculino	58 anos	3 meses	Peritonismo	Laparotomia infraumbilical	apêndice perfurado e peritonite	Alta após 30 dias

* FID- right iliac fossa

## DISCUSSION

Acute appendicitis is the most common cause of acute abdomen, with a peak incidence in the 2^nd^ and 3^rd^ decades of life. There is risk of 7% of a person presenting it during lifetime^3^^,^ 6. However, despite being relatively common condition in the general population, its appearance after liver transplantation is a rare condition, with few reports or studies in the literature; it has incidence ranging from 0.09% to 0.49% 3 ^,^ 11.

The recent increase in solid organ transplants, as well as better surgical, drug and higher survival rate of transplant patients conditions, tend to significantly increase the number of cases reported in literature^3^^,^ 7.

In liver transplant patients the cause of appendicitis is not different from that found in patients not immunosuppressed, being the main causes mechanical obstruction and bacterial overgrowth. In addition to these causes, there were already described in literature lymphoid hyperplasia and infections by cytomegalovirus^2^^,^ 3 ^,^ 7.

Clinical predominate is non-specific gastrointestinal symptoms, being appendicitis easily confused with other transplant complications^11^. Pain in the lower right quadrant is the most commonly reported symptom, and may constitute the only symptom^7^. Described symptoms are nausea, vomiting, fever and diarrhea^3^^,^ 7 ^,^ 11. In addition, some aspects are atypical, attributed to immunosuppressive therapy, increasing the difficulty of correct diagnosis^4^. In this study, the symptoms were consistent with the literature, since the pain was found in all patients, and the main symptoms were pain well located in the right iliac fossa (40%) and peritoneal irritation (40%).

Laboratory findings in this group may differ from the population not immunosuppressed, since most patients showed no leukocytosis (>10,000/mm³), a finding expected in appendicitis^5^. Laboratory results of liver and biochemical function usually are normal^7^. Absence of leukocytosis and unspecific laboratory tests delay diagnosis of the disease.

Despite that this is a condition whose diagnosis is eminently clinical; imaging studies are very useful both to correct diagnosis and to rule out other complications. Abdominal ultrasound examination is the most accessible, inexpensive and has good diagnostic accuracy when performed by an experienced operator. But, the choice method is computed tomography showing high sensitivity (94%) and specificity (94%)^3^^,^ 7 ^,^ 11.

The differential diagnosis is difficult; it is important to rule out other possible complications such as perforation of the bowel, biliary fistula, and other diseases related to the impairment of the graft, such as infections, vascular thrombosis and rejection^3^.

The diagnosis delay in this group is associated with a high incidence rate of appendix perforation and other complications, especially after 72 h of symptom onset. In patients not immunosuppressed, drilling rate varies according to the age, and the recorded incidence is 8-41.5%^1^. In four of five patients in the present study there was appendicitis complicated by suppuration (40%) or perforation (40%).

The treatment of choice is surgery, which should be held as early as possible. Most studies indicate appendectomy with conventional laparotomy incisions in the range 1-3 days of onset; this treatment timing was applied in all patients in the present study. Literature has described laparoscopic approaches successfully done and recommended its use in specific cases^2^^,^ 3 ^,^ 7.

With diagnosis delay and high rates of complications, hospitalization time is increased, being recorded average of 1-20 days^3^. In the population studied, the length of stay ranged from 2-45 days. The long hospitalization occurred in liver transplant recipients with wound complications.

## CONCLUSION

Acute appendicitis after liver transplantation is rare; the event is associated with high rate of drilling, due to delays in diagnosis and treatment, and consequent increase in hospitalization.
